# Magnetic resonance image tissue classification using an automatic method

**DOI:** 10.1186/s13000-014-0207-7

**Published:** 2014-12-24

**Authors:** Sepideh Yazdani, Rubiyah Yusof, Amirhosein Riazi, Alireza Karimian

**Affiliations:** Malaysia-Japan International Institute of Technology (MJIIT), Universiti Teknologi Malaysia, Jalan semarak, Kuala Lumpur, 54100 Malaysia; Control and Intelligent Processing Center of Excellence School of Electrical and Computer Engineering, University College of Engineering, University of Tehran, Tehran, Iran; Department of Biomedical Engineering, Faculty of Engineering, University of Isfahan, Isfahan, Iran

**Keywords:** Statistical segmentation, Magnetic resonance imaging, Image segmentation, Histogram-based segmentation method, SVMs, Brain tissue classification

## Abstract

**Background:**

Brain segmentation in magnetic resonance images (MRI) is an important stage in clinical studies for different issues such as diagnosis, analysis, 3-D visualizations for treatment and surgical planning. MR Image segmentation remains a challenging problem in spite of different existing artifacts such as noise, bias field, partial volume effects and complexity of the images. Some of the automatic brain segmentation techniques are complex and some of them are not sufficiently accurate for certain applications. The goal of this paper is proposing an algorithm that is more accurate and less complex).

**Methods:**

In this paper we present a simple and more accurate automated technique for brain segmentation into White Matter, Gray Matter and Cerebrospinal fluid (CSF) in three-dimensional MR images. The algorithm’s three steps are histogram based segmentation, feature extraction and final classification using SVM. The integrated algorithm has more accurate results than what can be obtained with its individual components. To produce much more efficient segmentation method our framework captures different types of features in each step that are of special importance for MRI, i.e., distributions of tissue intensities, textural features, and relationship with neighboring voxels or spatial features.

**Results:**

Our method has been validated on real images and simulated data, with desirable performance in the presence of noise and intensity inhomogeneities.

**Conclusions:**

The experimental results demonstrate that our proposed method is a simple and accurate technique to define brain tissues with high reproducibility in comparison with other techniques.

**Virtual Slides:**

The virtual slide(s) for this article can be found here: http://www.diagnosticpathology.diagnomx.eu/vs/13000_2014_207

## Background

Segmentation of brain volume in 3D magnetic resonance brain images has important clinical applications. Segmented brain in MR Images can be used in the visualization and quantitative analysis of anatomical structures. The segmentation of brain MRIs aimed to assign each voxel to a particular tissue class has received considerable attention. In clinical research on brain structures, the illustration of tissue size is an important aspect of therapy that should be accomplished accurately.

There is a body of research in which MRI segmentation is still supervised by experts on a slice-by-slice interactive input, which is a labor intensive and time consuming task. These manual techniques suffer from inter and intra observer variability [1,2].

Automatic three-dimensional (3-D) segmentation of MR Images according to tissue type at the voxel level is an important issue in many neuroimaging applications [3,4]. Changes in the composition of white matter (WM), gray matter [5], or cerebro-spinal fluid (CSF) in the whole brain volume or within special areas can be used to define disease entities and physiological processes or to determine disease severity [6-8].

In this paper, we proposed an automatic framework for brain segmentation into WM, GM and CSF. There has been a wide range of automatic segmentation methods proposed in the literature. The corresponding state of the art automatic techniques can be categorized by considering their methodological approach [9].

Classification techniques in the field of brain MRI data can be categorized into two groups: parametric and non-parametric algorithms.

Most of the parametric algorithms make the assumption that the intensity of three brain tissue types follows a Gaussian distribution. The statistical model parameters are generally estimated using the maximum a posteriori (MAP) or maximum likelihood [10] technique and the expectation maximization (EM) method is applied in the optimization process. The Expectation Maximization (EM) algorithm [11] iterates between the estimation of tissue class probability.

EM is in the category of histogram-based segmentation approaches. Histogram based techniques take into account the relative position of the valleys, peaks and other statistics extracted from the image histogram. The problem is that these techniques usually do not take into account the spatial information contained in the image [12].

In non-parametric statistical methods, information on the probability distribution for image segmentation is not required. Hall et al. applied a nonparametric model Fuzzy C-Means method for tissue classification [13]. Fuzzy C-Mean is a powerful method that has been used in MRI segmentation [14,15] in which voxels are partially segmented into various tissue classes using different memberships for each tissue type [16]. One of the main problems of the FCM methods is that the results are influenced by artifacts such as noise. Since MR images always include a considerable amount of noise, this leads to further degradation with segmentation.

Many researchers classified brain MR Images using an artificial neural network. In comparison with FCM, the FCM method was shown to be superior on normal brain and worse for abnormal brain with edema, tumor, etc. [17].

Machine-learning algorithms have proven to obtain acceptable results in many cases. The SVM algorithm is considered as a desirable candidate because of its high generalization performance without the need for prior information [18]. The SVM has attracted a high degree of interest amongst the machine learning research community [19]. Some studies have reported that the SVM is usually more able to deliver higher performance in terms of classification precision than the other classification methods. The SVMs do not suffer the limitations of data dimensionality and limited samples.

There is no unique algorithm for accurate brain MRI segmentation because each method has its disadvantages and drawbacks. As mentioned before some of the automatic segmentation methods are complex and some of them are not sufficiently accurate for certain applications.

For example the thresholding methods may fail to determine the right thresholds for brain tissues, especially the threshold for WM and GM. Random field methods require an energy function that is usually very difficult to determine that describes the drawback. These methods are also computationally intensive, which may prohibit their practical use [10].

Regarding FCM techniques, in the case of low quality images, which are corrupted by noise or bias field, the performance of FCM techniques strongly decreases. In the case of noisy images, anatomically erroneous structures may appear, for example single GM voxels within homogeneous WM. In the case of images corrupted by bias field, considerably sized clusters of voxels can be erroneously classified, leading to considerable errors in tissue estimation [20,21].

These problems cause inaccurate and weak results in human brain segmentation.

In this paper, support vectors, which are important for classification, are obtained by learning from the training samples in the last stage.

The key aspect of the proposed framework is that we combined three methods to have an accurate classification, each of which individually extracts a different set of constraints of the problem and the results of each step simplifies the one that follows it.

This has been demonstrated through experiments on both simulated and real data, where accurate and robust segmentation results can be obtained to find optimal segmentations. The rest of this paper is organized as follows:

In section Methods we present the new automatic framework for classification of brain tissues that combines three methods, each of which are more robust than its individual components, and other currently used methods. In Section Results and discussion, we present experimental results and discussion regarding verification of medical image segmentation. In this section we present a comparison of our results in a database of 18 simulated image volumes and 18 real images. The segmentation performance is evaluated for the proposed method. Section 4 contains concluding remarks.

The proposed integration technique is a fast and accurate approach to obtain optimal segmentations, given the intensity models that incorporate the spatial coherence information.

## Methods

In this study we proposed a hybrid of statistical- machine learning based segmentation method to segment three tissue classes (WM, GM and CSF) in MRI, where SVM is applied to improve the results.

Choosing proper features has a strong effect on brain MRI segmentation. The three main tissues of brain not only have various intensities, but also their intensity varies among different slices. In some slices, the voxel intensities of GM tissues are very close. Another possibility that should be considered is the existence of tiny regions from the skull stripping section. In some scans, the non-brain voxels have a similar intensity to GM, WM and CSF. Thus, identification of brain tissues according to the intensity features singly is not recommended. In this paper we used different features to have appropriate segmentation for all cases.

We integrated two types of information, MR intensity and voxel location information, as well as spatial relationships of voxels to improve the overall segmentation performance.

We first segmented the images using a statistical histogram based algorithm. Then we used 3D feature extraction technique for texture analysis in addition to spatial information to the classification process. Finally SVM classifier is used especially for the brain margin classification. In this step the classifier assigns a label of brain tissue using first-order statistics and other features that are extracted from the target area. In the proposed framework, the overall segmentation performance is improved by employing SVM.

**Figure 1:**
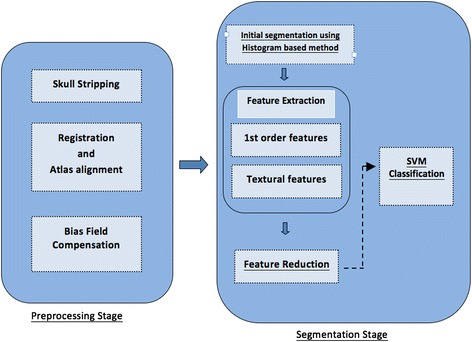
**General overview of the proposed technique.**

To compensate MRI artifacts the preprocessing steps are applied prior to actual segmentation, which are explained as follows. A general overview of our method is shown in Figure 1.

### Stage 1: image pre-processing

The purpose of this stage is to enhance and extract the non-brain tissues within the MRI. The output image is further segmented into white matter, gray matter and CSF. To enhance the segmentation process, we used four steps for preprocessing stage, which are explained as follows.

**Step 1: Skull Stripping**

Firstly, the T1-weighted brain images were brain-extracted applying the FSL default BET brain extraction process, which removes any non-brain tissue such as such as fat, bone, skin, and muscle from the image using the FAST4 tool [22].

**Step 2: Registration and atlas alignment**

In this step we performed a spatial registration of the input scans. Image registration is the act of aligning scans to related corresponding features. For most types of image processing on more than one image, it is required that the images are aligned. Therefore one voxel position displays the same anatomical position in all MRI scans. The second step applied a standard 12 degrees of freedom registration to the template.

**Step 3: Bias Field Compensation**

Bias field is also called intensity inhomogeneity, which is one of the main problems and challenging issues in MRI. Intensity inhomogeneity is caused by the fact that it is technically impossible to create a completely uniform radio frequency magnetic field [23].

Bias field has a negative effect on segmentation results and prevents description of voxel tissue content based exclusively on image intensity [24]. Consequently quantitative analysis of MR images requires bias field correction [25]. Different methods exist to compensate for the inhomogeneity problem.

In this paper to compensate bias field we applied a nonparametric intensity non-uniformity normalization (N3) method. This is an iterative method that estimates both intensity distribution of true tissue and multiplicative bias field. N3 method is accurate, robust and automatic and makes no assumptions about the type of anatomy in MR image [26].

The assumption in the N3 method is that nonuniformity blurs the histogram of image in a way that it can be identified and removed. This blurring distribution is referred to as the blurring kernel F. The basis of the N3 algorithm is presented as follows:

1 $$ \upsilon \left(\mathrm{x}\right)=u\left(\mathrm{x}\right)\kern0.15em f\left(\mathrm{x}\right)+\left(\mathrm{x}\right) $$

Let x, v and u be the location, measured signal and true signal emitted by the tissue respectively. f is an unknown bias field or intensity inhomogeneity and n is the additive noise assumed to be independent of u. For bias field correction, the first step is estimating its distribution.

Due to the simultaneous presence of n(x) and f(x) it is difficult to solve the problem. Thus, a common solution is to neglect the additive noise. For the two-dimensional discrete image case and using a log transform, the bias field is made additive.

This model can be simplified by neglecting the noise and taking the log of both sides, therefore it becomes additive rather than multiplicative. Instead of v, u, f, we deal with log v, log u, log f, then the formation model becomes additive:

2 $$ \log v\ \left(i,\ j\right) = \log \kern0.15em u\ \left(i,\ j\right)+ \log\ \mathrm{f}\left(i,j\right) $$

Let V(*u,v*), U(*u,v*) and F(*u,v*) present the probability densities of *v(i,j )*, *u(i,j)* and *f(i,j)* respectively. Eq. 2 can be expressed as:

3 $$ \mathrm{V}\ \left(u,\ v\right) = \mathrm{U}\ \left(u,\ v\right) + \mathrm{F}\ \left(u,\ v\right) $$

Making the approximation that ln *u* and ln *f* are uncorrelated random variables, Eq. 3 is found by convolution as follows:

4 $$ \mathrm{V}\ \left(\widehat{\upsilon}\right) = F\left(\widehat{\upsilon}\right)\kern0.15em *\kern0.15em U\left(\widehat{\upsilon}\right) $$

The multiplication corrupts the field and a division can undo the corruption. In the frequency domain, multiplications and divisions are converted to convolutions and deconvolutions as follows:

5 $$ \mathrm{V}\ \left(\widehat{v}\right) = \mathrm{F}\left(\widehat{v}\right)\times \mathrm{U}\left(\widehat{u}\right)=\mathrm{F}\left(\widehat{v}-\widehat{u}\right)U\left(\widehat{u}\right)d\left(\widehat{u}\right) $$

in which V, U, and F are probability densities. After this stage the uniformity distribution (F) is modeled and viewed as blurring intensity distribution U that is the main stage for correcting bias field.

In this paper we used the idea of singularity function analysis (SFA) [25], which assumes that anatomical information of MRI occurs in the high spatial frequencies in the image. In this method, after low pass filtering, since useful low spatial frequencies are removed during the filtering process, the filtered version of the ideal signal does not look like the original unbiased signal. SFA models recover the removed low frequency information via reconstructing spatial information from the remaining high spatial frequencies. The output of this step is an intensity non-uniformity corrected MR image.

### Stage 2: Brain segmentation

In this paper a fully automatic framework for segmentation of brain MR Images is proposed for brain tissue segmentation in the magnetic resonance images. This framework is a new combination of three techniques that includes three main stages to have an effective tool. Each stage individually exploits gray level, textural information, spatial information and relationship with neighboring voxels in the images. The first step provides an intensity-based segmentation of images into different tissue types. To produce a much more efficient segmentation technique further processing steps are required for several reasons such as:

- The brain voxels in MRI, especially at the margins and edges, are not defined by the unique intensities and these images have overlapping tissue boundaries.
- Histogram based methods do not take into account the spatial information contained in the image.
- Intensity similarity between CSF and WM or CSF and GM make the segmentation process problematic.

Consequently further processing is also needed after the first step to have an accurate and robust segmentation. Thus we used SVM, especially for the brain margin classification and segmentation enhancement.

#### Brain tissue segmentation based on 3D histogram based technique

The histogram-based brain segmentation method consists of three stages. The first stage is background/foreground thresholding to separate the background and foreground tissues. The second stage is disconnecting brain from nonbrain tissues. The third stage is segmentation of brain into three tissue classes.

- Background/foreground thresholding

**Figure 2:**
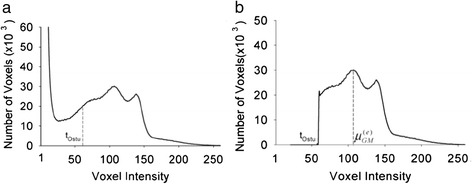
**Histogram of the image p(i). (a)** Histogram of the real data p(i) The Ostu threshold, which segments the background from foreground the voxels, is marked. **(b)** Histogram of the same data as in **(a)** but after thresholding the background/foreground voxels.

The threshold (tOstu) was defined based on an analysis of the image histogram [27,28]. As shown in Figure 2(a), the histogram of a real MRI image is presented. Any voxels with signal intensity lower than a defined threshold have been removed. These removed voxels included those contributed by very low-intensity components. The histogram of resultant image (I_1_) is demonstrated in Figure 2b, in which the low-intensity peak in Figure 2(a), has been deleted and the peaks corresponding to the WM (peak at right) and GM (middle peak) remain.

- Brain extraction from skull and other had tissues

This section is based on two assumptions: The first one is that brain region is connected to the skull and neck tissues through infrequent connections in the MR image. Most of these connections present a lower intensity than GM intensity, but a small portion has a similar intensity. The second assumption is that the brain is the largest connected component in the head MR image. The brain is extracted from the skull by the following steps:

1. Three peaks in the image histogram are searched and located. Based on the assumption that each of the tissues has a Gaussian distribution, mean intensity value of second peak or GM is μ_GM._
2. The binary mask image is created from image I_1_ using a threshold (t_m_) at a level lower than μ_GM._
3. A morphological binary opening is used to binary mask applying a spherical structuring element with a radius of three voxels. This stage disconnected the brain from the skull and other tissues.
4. In this stage we carried out a connected component processing on the resultant images. The largest connected component (brain) is kept and the remaining components (non-brain tissues) are removed.
5. Three steps of dilation are performed from the last step applying a spherical structuring element.
6. The extracted brain images (I_2_) is created by voxel-to-voxel multiplication of I_1_ image and the resultant mask images (M_1_).

6 $$ {\mathrm{I}}_2 = {\mathrm{I}}_1.\ {\mathrm{M}}_1 $$

- Segmentation of brain into three tissue classes

Based on the gray level analysis, it can be assumed that tissues of brain image belong to one of four classes, which follow a normal distribution.

- C1: Background, noise and cerebro-spinal fluid.
- C2: GM. It forms the central peak in the histogram.
- C3: WM. It forms the peak on the right side of the histogram.
- C4: Other tissues with high gray value.

**Figure 3:**
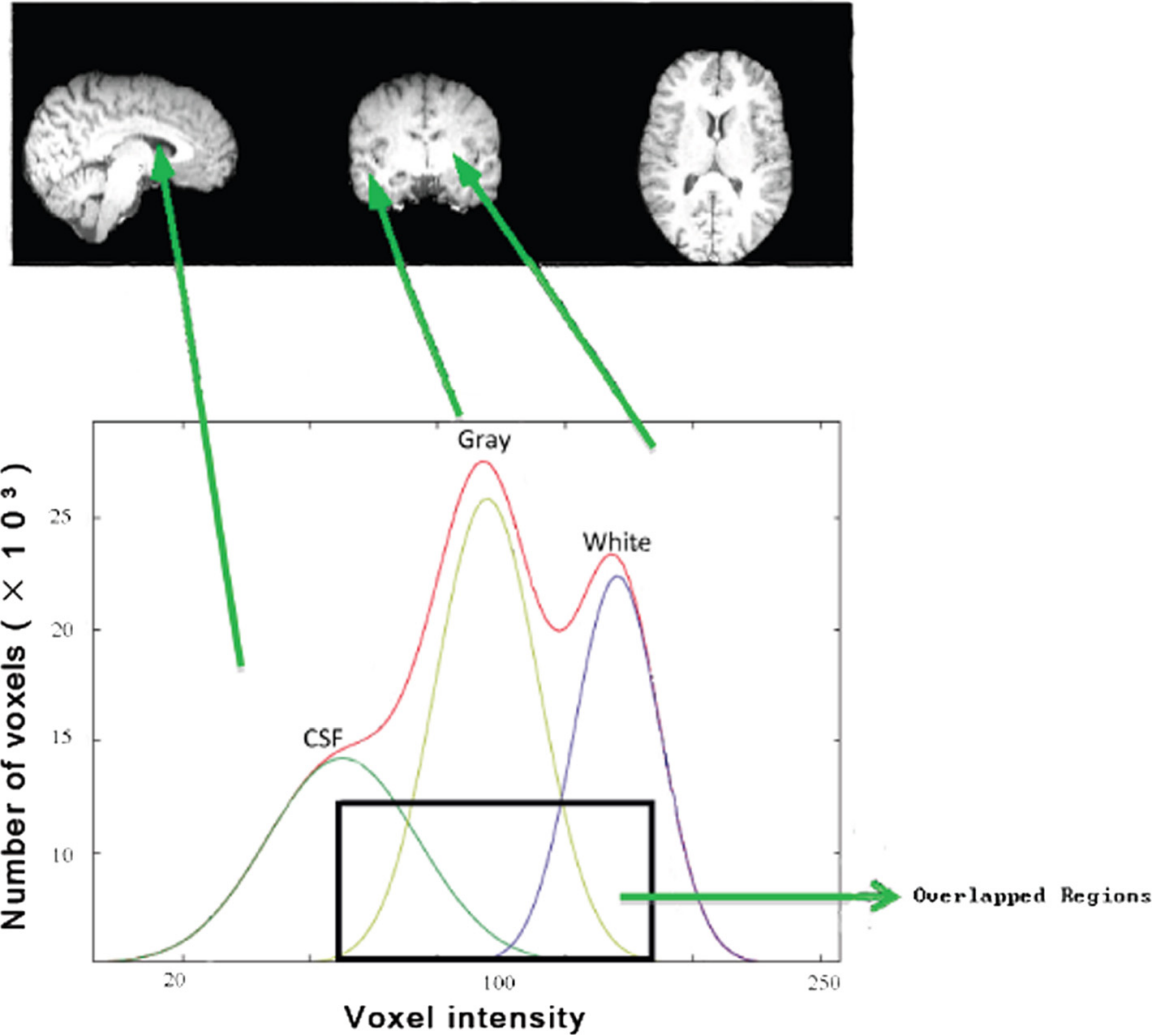
**Approximated histogram by Gaussian function (red line).** The approximated histogram is the sum of the estimated normal distribution of gray levels of C1 (green line), C2 (yellow line) and C3 (blue line).

An approximated histogram in Figure 3 is created modeling these different classes with Gaussians. Since class C4 has very few voxels, only classes C1, C2, and C3 are modeled.

Accordingly, the approximated histogram is presented in Eq. 7. The equation presents the probability of each voxel belonging to each of the tissues:

7 $$ p\hbox{'}\left(i;\kern0.15em v\right)=\underset{k=1}{\overset{3}{\varSigma }}{p}_k \exp \kern0.15em \left(-\frac{1}{2}{\left[\frac{i-{\mu}_k}{\sigma_k}\right]}^2\right) $$

Let *i* and *pk* be the gray level and probability of a voxel of tissue class *k = {1, 2, 3},* to acquire intensity *μk. μk* is the mean gray level of tissue class *k, σk* is the standard deviation of the Gaussian function. Its variance is therefore *σ*2. *σk* demonstrates the class *k* and *v = (pk, σk, μk) is* the vector of parameters of the Gaussian functions. *P*′*(i; v)* is the probability that a voxel has intensity *i*, using the vector of parameters *v.* Therefore, the values μk should correspond to the main peaks in the image histogram. The parameters of the Gaussian functions are adjusted so that *p*′*(i; v)* fits the image histogram. In this step, to have maximum similarity between original histogram and estimated histogram some of the parameters are re-estimated using Eq. 8. Therefore, the vector of optimal parameter v* = (pk*, σk *, μk *) is:

8 $$ {v}^{*}=\underset{v}{ \arg \min}\underset{i=0}{\overset{W-1}{\varSigma }}{\left[p(i)-p\mathit{\hbox{'}}\left(i;\kern0.15em v\right)\right]}^2 $$

In the next step we used Levenberg–Marquardt that is a recursive method to estimate an optimal kernel. The kernel is used to minimize differences between estimated distribution and original one. The minimization process is performed using Eq. 8.

Let W be the number of gray levels of histogram. The initial vector of parameters for the minimization step is based on kernel density estimation, which is a method used to estimate the probability density function of random variable. This variable is the image histogram, p(i). Therefore, the kernel density estimation is:

9 $$ \widehat{p}\left(i;\kern0.15em h\right)=\frac{1}{Nh}\kern0.15em \underset{j=0}{\overset{W-1}{\varSigma }}p(j)K\kern0.15em \left(\frac{i-j}{h}\right) $$

Let h and K be the bandwidth parameter of the kernel, and the kernel function respectively. The parameter j is the internal variable of the summation over the W gray levels [27,28].

Since the C1 class does not always display a peak to compute the initial vector of parameters to adjust p = (*i; v*), the best way is defining the peaks of C2 and C3. Figure 2 displays the real image histogram and Figure 3 shows the approximated histogram, p = ′(i; v) (red line) is created by the sum of the functions displaying the gray level distributions of three class CSF (green line), GM (yellow line) and WM (blue line).

However, due to the intensity similarity between CSF and WM or CSF and GM the initial segmentation step is not enough to have a robust and accurate classification. Inevitably, overlapping is also occurring in these images (Figure 3). To compensate for these problems we need further processing steps. In the next step we will extract some textural features from the image and finally use SVM to improve the classification process.

#### Feature extraction

The purpose of feature extraction stage is to decrease the original data set by extracting most important features. Choosing optimal features has a strong effect on segmentation results.

The most prominent features for classification of pathologic and healthy tissues are the image intensities, which are used in the initial classification. The problem is that the intensity of constructing brain tissues varies among different slices. In some slices, the intensity of different tissues is similar. In some cases, the non-brain voxels have a similar intensity to WM, GM and CSF. As using intensity information as the only feature is not sufficient to have an accurate segmentation, a set of features must be considered. Therefore we carried out texture analysis for describing texture of the images to have adequate features for accurate segmentation.

We extracted different features such as first and second order texture information to have accurate tissue segmentation for all cases. First-order textures can be computed fast and easily from small patches around each voxel in all four modalities.

The extracted features prepare the specification of the input data to the classifier by considering the definition of the relevant characteristics of the brain image into a feature space. The classifier assigns a label of brain tissue using different features, which are extracted from a VOI.

**Figure 4:**
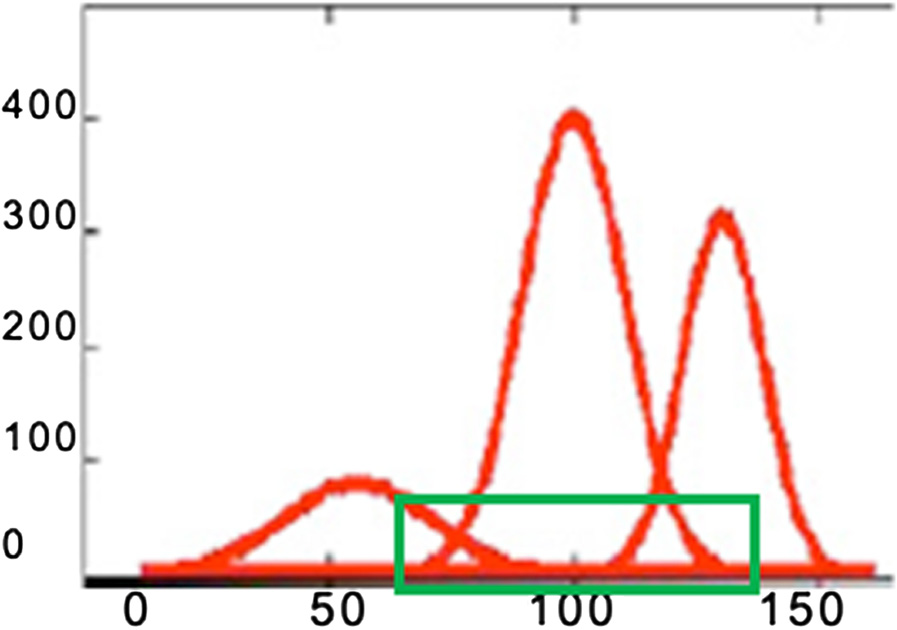
**Target area or input data for 3-D GCM.**

In this paper, the statistical features based on image intensity and other features which are extracted from 3-D gray level co-occurrence matrix (GCMs) are used to describe texture differences of complex images in the spatial relationships of voxels. In this section each overlapped voxels and their 18-connected voxels are used as input for GCM that is an improvement stage. The target area is demonstrated in Figure 4.

The GCM is a well-established approach for describing the spatial distribution that includes second-order statistics of gray levels.

GCM prepares information on how often a gray level occurs at different directions and determines the joint probability density of the occurrence of gray levels in direction Φ and specified distance d from each other [21].

**Figure 5:**
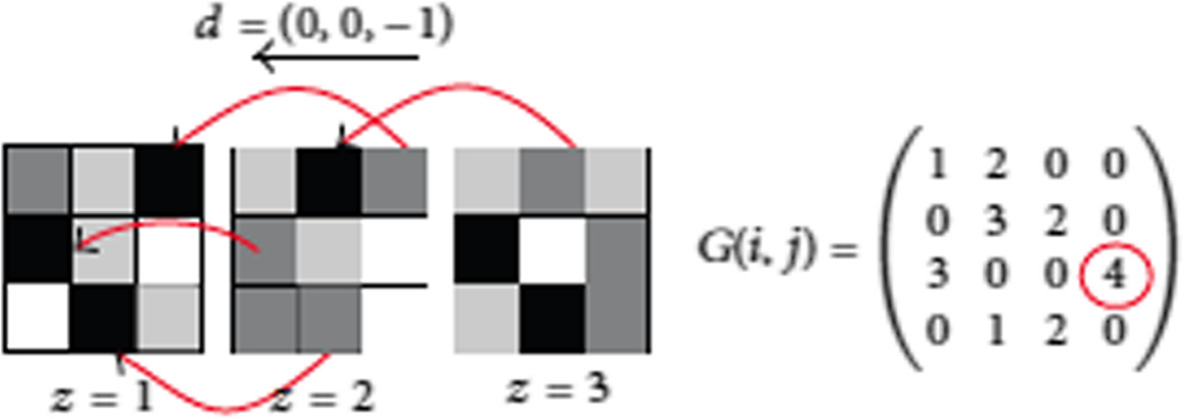
**3D GCM calculation in direction (0, 0, −1).** z values present different slices (z coordinate). Arrows indicate the relationship between voxels for computing the co-occurrence value in the direction presented.

To extract useful information an interested voxel and some of the neighbors are selected as a window. The mentioned window is applied for all of the voxels in the image. Required information is finally extracted from the windows. In 3-D image we have a cube instead of 2-D window (Figure 5) [21]. Eq. 10 is used to define the mentioned cube.

10 $$ \begin{array}{l}{G}_d^{\phi}\kern0.15em \left(i,\kern0.15em j\right)\\ {}\underset{z=1}{\overset{V_z-{d}_z}{\varSigma }}\underset{y=1}{\overset{V_y-{d}_y}{\varSigma }}\underset{x=1}{\overset{V_x-{d}_x}{\varSigma }}\left\{\begin{array}{l}1,\kern1em \mathrm{if}\\ {}\kern2em \left(\mathrm{Q}\kern0.15em \left(x,\kern0.15em y,\kern0.15em z\right)=i\right)\\ {}\kern3.5em \wedge \Big(\mathrm{Q}\kern0.15em \left(x+{d}_x,\kern0.15em y+{d}_y,\ z+{d}_z\right)\\ {}=j\Big),\\ {}0,\kern1em \mathrm{otherwise}\end{array}\right.\\ {}\kern23.5em i,\kern0.15em j=,\kern0.15em .\kern0.15em .\kern0.15em .\kern0.15em ,N,\end{array} $$

N is the number of gray levels in the image considered for GLCM calculation and *v = (v*_*x*_*, v*_*y*_*, v*_*z)*_ is the position of the voxel. *d = (d*_*x*_*,d*_*y*_*,d*_*z*_*)* is the distance in each direction.

In this paper, we considered cubes of size 21 × 21 × 21 instead of a square window. Choosing the window size is one of the important issues, as it can determine the discrimination capabilities of the extracted features. A small window reduces the computational burden. Furthermore while large windows capture textural characteristics, they increase the processing memory and requirement.

Figure 5 demonstrates an example of 3D GCM for *N* =4 gray levels and *d* = (0, 0, −1) direction. In this figure, z values show z coordinates (various slices). The arrows demonstrate the relation between voxels for computing the co-occurrence value in the direction displayed [21].

In this study, for each distance thirteen co-occurrence matrix features are computed from a 21 × 21 × 21 sliding window in the brain image as follows: contrast, angular second moment, variance, correlation, sum average, inverse different moment, sum entropy, sum variance, difference variance, entropy, difference entropy, information measure of correlation 1 and 2.

In addition, two first order texture features (standard division and mean) of each feature over the 13 co-occurrence matrices are computed, including 26 GCM-based features for each distance. In general 130 features were calculated for per Voxel Of Interest (VOI).

#### Feature reduction

Even a modest GCM algorithm can create many more textural features than are appropriate for the number of cases that will be subjected to segmentation. The amount of data for testing and training affects the testing and training process time of the next section (SVM). Thus it is ‪desired to apply the minimum amount of data essential to produce comparable classification performance. To solve the problem of large data sets we decreased the number of the training data set while preserving the classification performance. In this paper Stepwise Discriminant Analysis (SDA) method is used which is a statistical technique to decrease feature dimension. After feature selection stage the subset of features has been applied to process the images to provide the segmentation applying the last step (SVM). We used support vector machines especially for brain margin classification and segmentation improvement.

#### Segmentation enhancement by SVM

The support vector machine classifier is considered a well-defined candidate for three main reasons [5,29].

- Support vector machines work well for classifying objects that are not linearly separable.
- SVMs have good generalization ability without the need for priori knowledge even if the dimension of the input space is very high.
- We can also combine or replace support vector machines with other classification techniques to obtain better segmentation results.

SVMs are currently the state-of-the-art technique to solve binary classification problems. They have shown good results in the literature for different pattern recognition tasks [30]. Due to the generalization ability, the SVM has accomplished great success in different applications. In this study we used SVM to enhance the segmentation process, to rank computed features from the extracted regions and to classify brain borders and overlapped regions.

After initial segmentation, to assign a label of each overlapped voxels a support vector machine classifier is used. SVM is trained for each tissue type based on the extracted features from the previous section. In this section the SVM approach is briefly explained.

**Figure 6:**
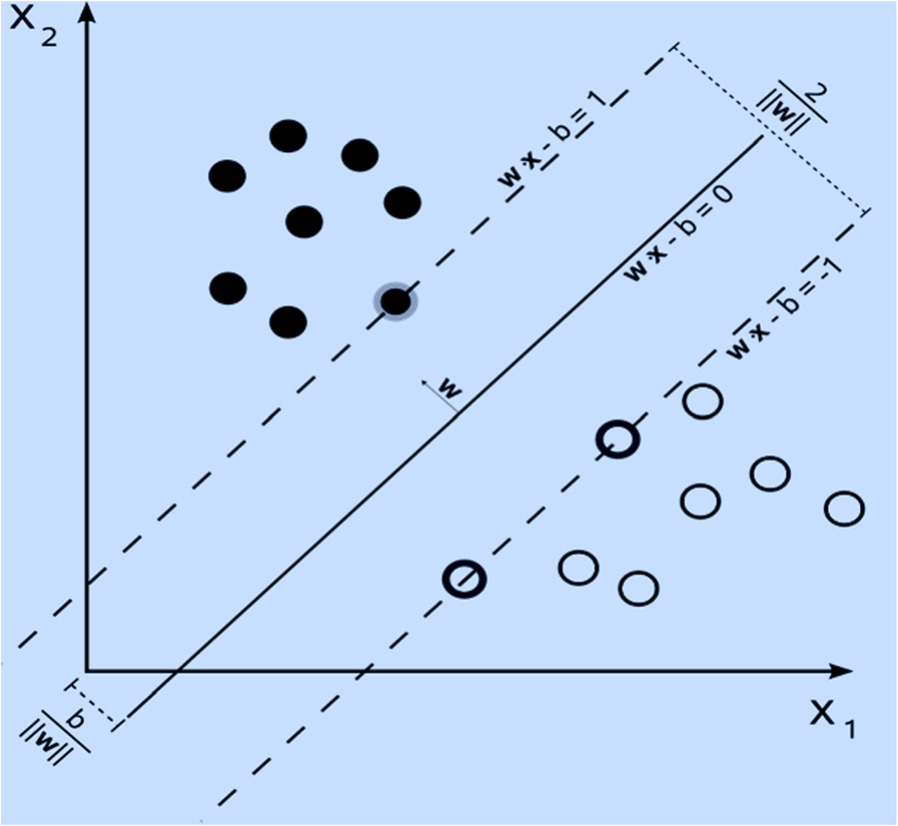
**SVM classification.** NeuroImage.

The SVM classifiers have a training step to determine a separating hyperplane for the data in the feature space. For a given training dataset, while there can exist different hyperplanes that maximize the separating margin between the two tissue classes, the SVM is based on the hyperplane that maximizes the separating margin between different classes (see Figure 6).

The hyperplane (f (x)) that classified the given dataset is defined as:

11 $$ f\left(\mathrm{x}\right)={\mathrm{w}}^{\mathrm{T}}\kern0.15em \varPhi \left(\mathrm{x}\right)+b $$

Following nonlinear transformation, the parameters of the f(x) are defined through the following minimization:

12 $$ \underset{\mathrm{w},\ \mathrm{b},\ \upxi}{ \min}\kern0.15em \frac{1}{2}{\mathrm{w}}^T\mathrm{w}+C{\displaystyle {\sum}_{i=1}^1{\xi}_i} $$

13 $$ \mathrm{subject}\ \mathrm{t}\mathrm{o}\ {y}_i\kern0.15em \left({\mathrm{w}}^T\phi \left({\mathrm{x}}_i\right)+b\right)\ge 1-{\xi}_i,\kern0.15em \mathrm{with}\kern0.15em {\xi}_i\ge 0,\kern0.15em \mathrm{i}= 1,\kern0.15em .\kern0.15em .\kern0.15em .\kern0.15em ,m. $$

Let c and w be the penalty parameter for the error term and the normal vector of the separating hyper plane respectively. W^T^*W maximizes the border that is around the decision function. ${}^C{\varSigma}_{i=1}^N\kern0.15em {\xi}_i$ minimizes the amount of training samples that lie within the mentioned margin. φ (x_i_) is the non-linear transformation that maps the samples into a higher-dimensional feature space. b is the offset of the hyper plane and (x_i_, Y_i_) are the pairs of the dataset. The appealing specification of this approach is that they offer the possibility to apply a kernel function (K (x_i_, x_j_) = φ (x_i_)^T^ φ (x_j_)) to transform the data into a higher-dimensional feature space. The kernel causes the data to be linearly separated through a maximum margin.

In other words the dataset with linear separability can be analyzed with a hyper plane, and the linearly non-separable dataset can be analyzed with the kernel functions. Different types of kernels are used in the literature, among which the most common are Gaussian radial-basis and polynomial functions. In this study, we used radial basis function (RBF) kernel for parameter selection of SVM classifier.

For soft margin classification Slack variables (ξi) are used. The non-negative slack variable (Eq. 13) always yield conceivable solutions by relieving the constraint of maximum margin. For multi-class classification, the classifier is extended by one-against-the others strategy. In this study an iterative labeling of neighboring voxels in the brain margins is performed applying the SVM classifier.

In this paper the training process is performed in two steps. In the first stage we extracted optimal features from each subject and then we trained each subject individually. We used overlapped and some of the non-overlapped voxels randomly as training data and overlapped voxels as test data.

In the second stage, we used all subjects for training process to have an accurate and robust classifier. In this section 10 samples of T1-weighted images of BrainWeb and 10 samples of IBSR datasets were applied for training. 7 subjects are applied as training dataset and 3 subjects to test the performance of the training process in each dataset.

For SVM training we fixed C (the penalty term for misclassifications) to 100. The classifier is trained for 10 000 samples per training image that are randomly extracted from the provided brain mask. With a proper selection of metric within the RBF kernel, the leap in implementation process did not occur. Laplacian RBF kernels reduce the Gaussian RBF error rate from 30% down to less than 10%. This improvement is because of the selection of a suitable metric and the proper generalization of SVMs.

## Results and discussion

Experiments have been performed on real images from IBSR and simulated images from Brainweb images as described below:

### Simulated image classification

The proposed framework has been first evaluated on simulated brain images. Since the simulated images of BrainWeb dataset have different noise levels (0%, 1%, 3%, 5%, 7% and 9%) and different intensity non-uniformity (bias fields) levels (0%, 20% and 40%), the evaluation of proposed method for accuracy and robustness in different noise and bias field levels is reliable and comparable. In addition knowing the ground truth we can have a quantitative verification of the performance of the several algorithms.

BrainWeb is a dataset which provides simulated brain MRI for different acquisition modalities such as T1, T2, etc. which are available at (http://www.bic.mni.mcgill.ca/brainweb/) [31,32]. A combination of different noise levels and intensity non-uniformity gives 18 simulated image volumes having voxel dimension of 1.0 × 1.0 × 1.0 mm.

**Figure 7:**
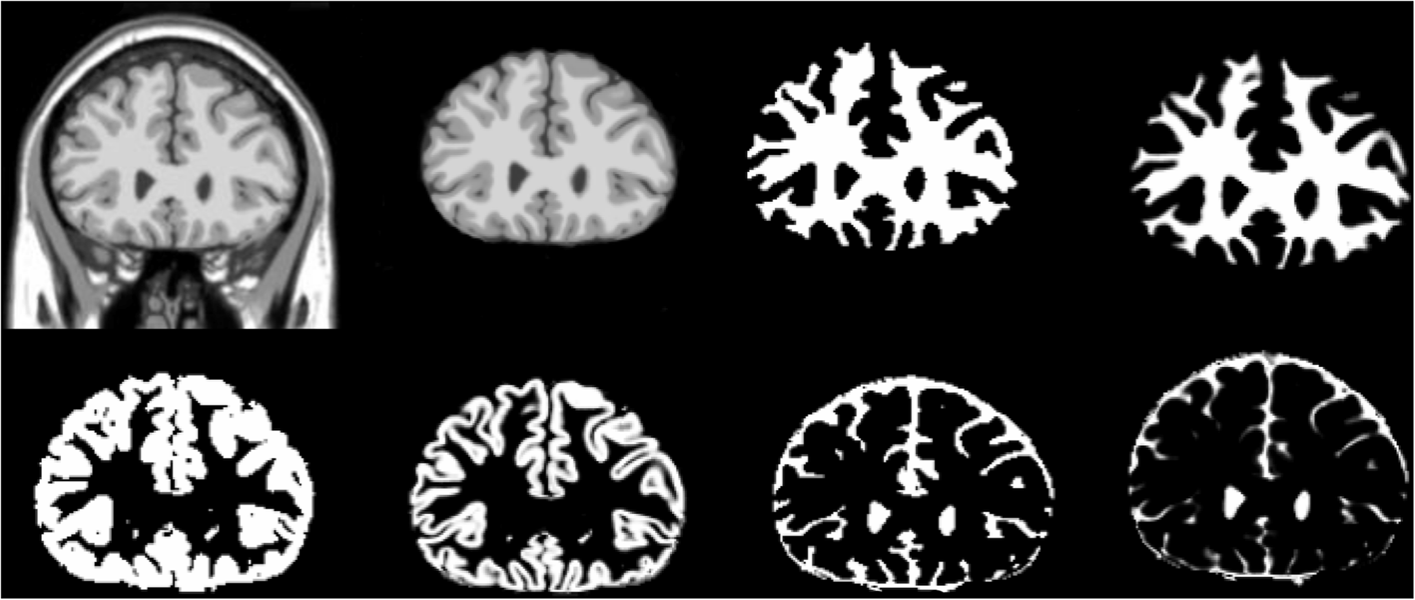
**Results of segmentations on the simulated images fom Brainweb, left to right, top to bottom; simulated image, Extracted brain image, Estimated WM, ground truth of WM, Estimated GM, ground truth of GM, Estimated CSF, ground truth of CSF.**

Each MRI is provided with an anatomical model that prepares main tissue class label for each voxel. For the method, the considered BrainWeb scans had been selected with classical acquisition parameters by considering T1-weighted scans with 1 mm resolution. The echo time has been fixed to 10 ms and the repetition time is 18 ms. In this paper, we applied 18 simulated scans of 181*217*181. For both the ground truth labeling and our labeled results, we obtained three-class labeling (see Figure 7).

For quantitative evaluation the kappa coefficient is calculated for white matter and gray mater tissues for each scan compared to anatomical model (ground truth) [33].

### Real image classification

The proposed method is also applied to real images from IBSR dataset, which are available at http://neuro-www.mgh.harvard.edu/cma/ibsr/. The IBSR has supported a collection of eighteen T1-weighted real MRI volumes that have been corrected by the IBSR for intensity non-uniformity.

These image datasets and their expert segmentations are available at http://www.cma.mgh.harvard.edu/ibsr/. The dataset is a set of eighteen 1.5 tesla 3D brain MR Images with their expert segmented images. The size of scans is 256 × 256 × 128 voxels and their resolution varies from 0.8 × 0.8 × 1.5 mm to 1.0 × 1.0 × 1.5 mm.

**Figure 8:**
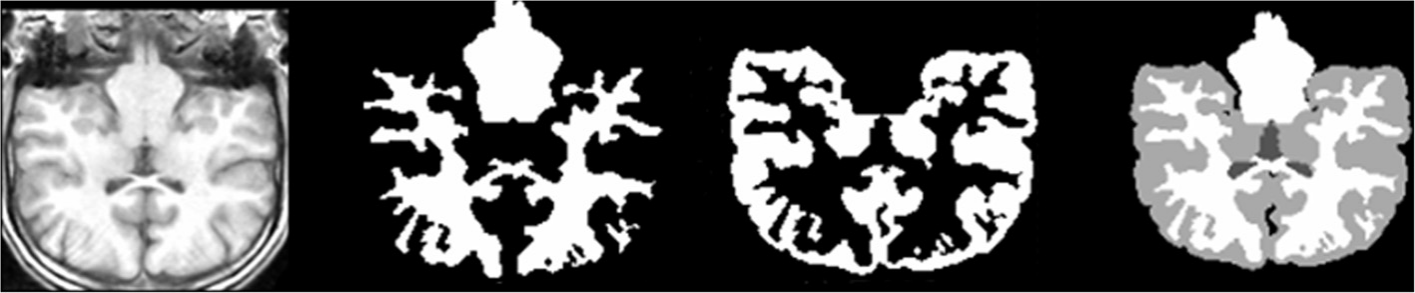
**Proposed algorithm applied to real database.** From left to right; a brain MRI slice of IBSR Database, Estimated WM, Estimated GM, Expert segmented.

In Figure 8, the results of the proposed method and the expert segmented volumes are demonstrated.

**Figure 9:**
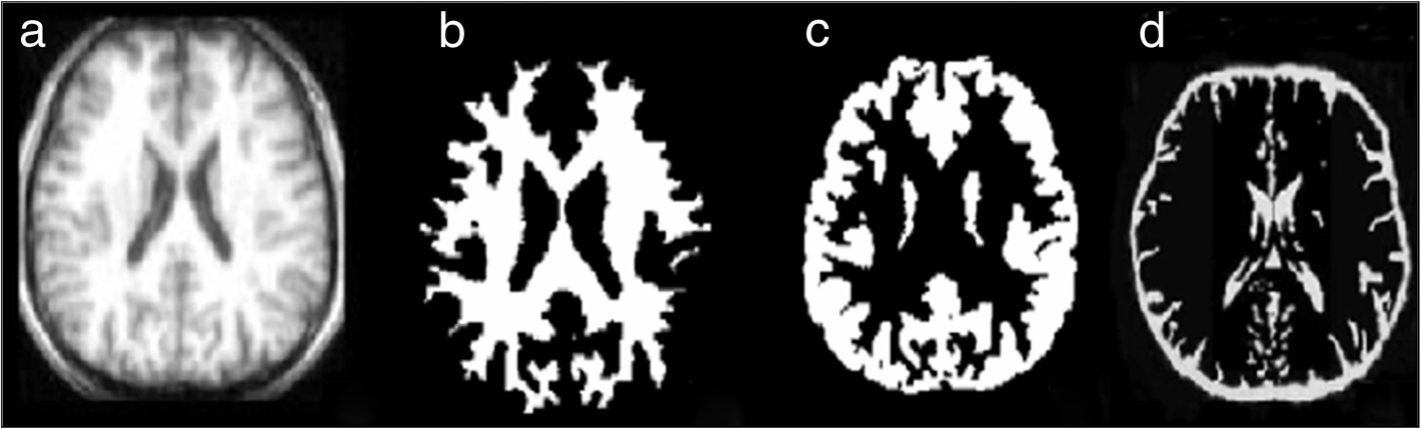
**Proposed algorithm applied to real database.** From left to right; a brain MRI slice of IBSR Database, Estimated WM, Estimated GM, Estimated CSF.

Dealing with real brain images, we faced different problems using BSE to segment the brain from non-brain tissues. Indeed, some non-brain voxels still appear in the scans, reducing the precision of segmentation results. In order to compensate for this problem we applied the atlas to separate brain and non-brain tissues and voxels where the atlas represents a zero probability of being WM, GM and CSF were removed. Figure 9 indicates the results of segmentation technique on the T1-weighted IBSR image.

### Experimental validation

To verify the performance of proposed method we performed two sets of experiments on real and simulated data. These data sets were selected because they have various levels of artifacts and they have also been used in published studies.

Since in these cases the ground truth (expert segmentation or anatomical model) is accessible, it is possible to have a quantitative evaluation of the performance of the technique.

In different studies in the literature the standard Jaccard similarity index is computed. This metric compares the similarities between the two sets as the ratio of the amount of their intersection divided by the amount of their union [4].

The other metric usually used to measure the set similarity is the kappa coefficient [34] that is determined as Eq. 14.

14 $$ k\left(S1,\kern0.15em S2\right)=\frac{\left|S1\cap S2\right|}{\left|S1\kern0.15em \mathsf{U}\kern0.15em S3\right|\kern0.15em \hbox{-} 1/2\left(\left|s1\backslash s2\right|+\Big|s2\backslash s1\right)} $$

K- index is used frequently in the literature and and has shown that it is appropriate for evaluation of image segmentation technique [33]. In this study, the kappa index is defined for both real and phantom datasets and the results are presented in the next sections.

#### Evaluation with the real dataset

The IBSR dataset usually used brain MRI for the validation of brain segmentation. The results of the mentioned method would be compared to those acquired by the other state of the art algorithms, specifically the following ones: statistical parametric mapping (SPM 5) [35], Expectation-maximization (EM) [36,37] , Hidden Markov Chains (HMC) [37], Fuzzy C-Means (FCM) [38,39], Non-Local Fuzzy C-Means (NL-FCM) [20,21,40].

Based on these considerations, the overlap measures are computed for WM and GM and the results obtained in the 18 scans are compared to the ones of these other algorithms. Because the images in the IBSR are segmented only into pure tissue types, our results are converted into three classes (GM, WM and CSF). Since the IBSR ground truth includes only internal CSF while our technique also defined sulcal CSF, we do not report results for CSF.

**Figure 10:**
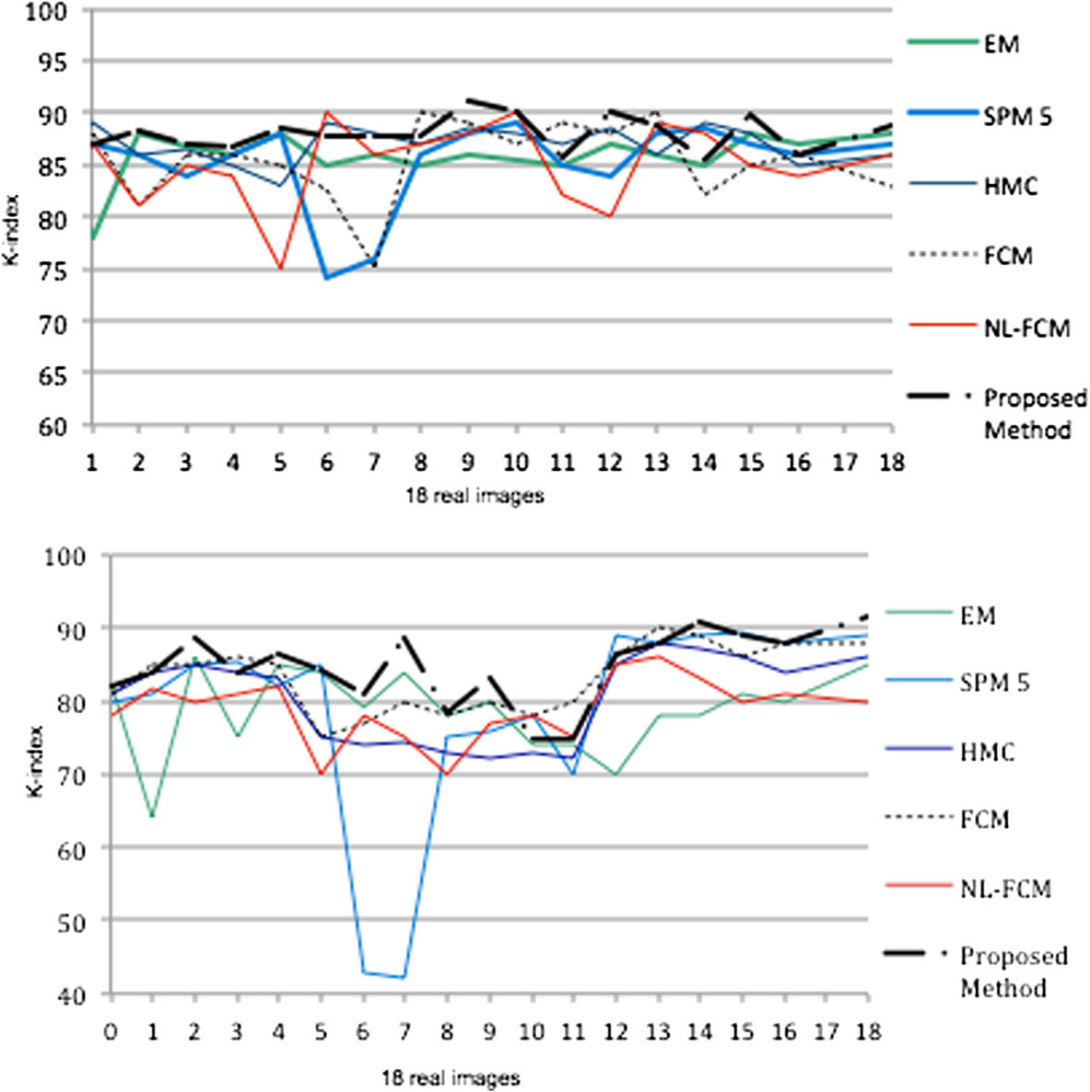
**Application of different segmentation methods through real images of IBSR database.** Top to bottom: overlap rate (K-index) of WM, Overlap rate (K-index) of GM.

Figure 10 presents the K similarity index (overlap rates) for the 18 real volumes on the IBSR database. Moreover, a comparison with other segmentation techniques is presented. The overlap rates of other methods in Figure 10 are based on free available reference software and published papers for brain segmentation in MRI.

The higher K index shows the best results and in most of the images in Figure 10 the k-index graph of proposed method presents higher values than other methods. When considering the results, using the proposed framework globally leads to better results than the other state of the art algorithms.

In comparison with currently used methods, the K-index of white matter is slowly improved in real images and the K-indexes of gray matter tissue displays that the improvement is significant. Gray matter graph is inherently much more tortuous than white matter. On average, our framework outperforms other competing methods in segmentation of WM and GM voxels. The mean K-indexes of IBSR scans for white matter are: SPM 5 = 85.30, EM =86.01, FCM =85.21, NL-FCM =84.83, HMC =86.91 and the proposed-technique =88.09. The mean K-indexes of gray matter segmentation are: SPM 5 = 74.93, EM = 74.47, FCM =78.91, NL-FCM =74.82, HMC =76.18 and the proposed-technique =80.35.

Quantitative mean and standard deviation results of 18 real images from IBSR dataset are demonstrated in Table 1 while results for other technique are also displayed.

**Table 1:** **Quantitative mean K-index and standard deviation results of 18 real images from IBSR dataset**

**Methods**	**White matter (%)**		**Grey matter (%)**	
	**Mean**	**Standard deviation**	**Mean**	**Standard deviation**
EM	86.01	2.24	74.47	5.57
SPM	5 85.30	3.90	74.93	13.88
HMC	86.91	1.67	76.18	5.81
NL-FCM	84.83	3.87	74.82	4.26
FCM	85.21	3.74	78.91	4.43
Proposed-Method	88.09	1.58	80.35	4.01

**Figure 11:**
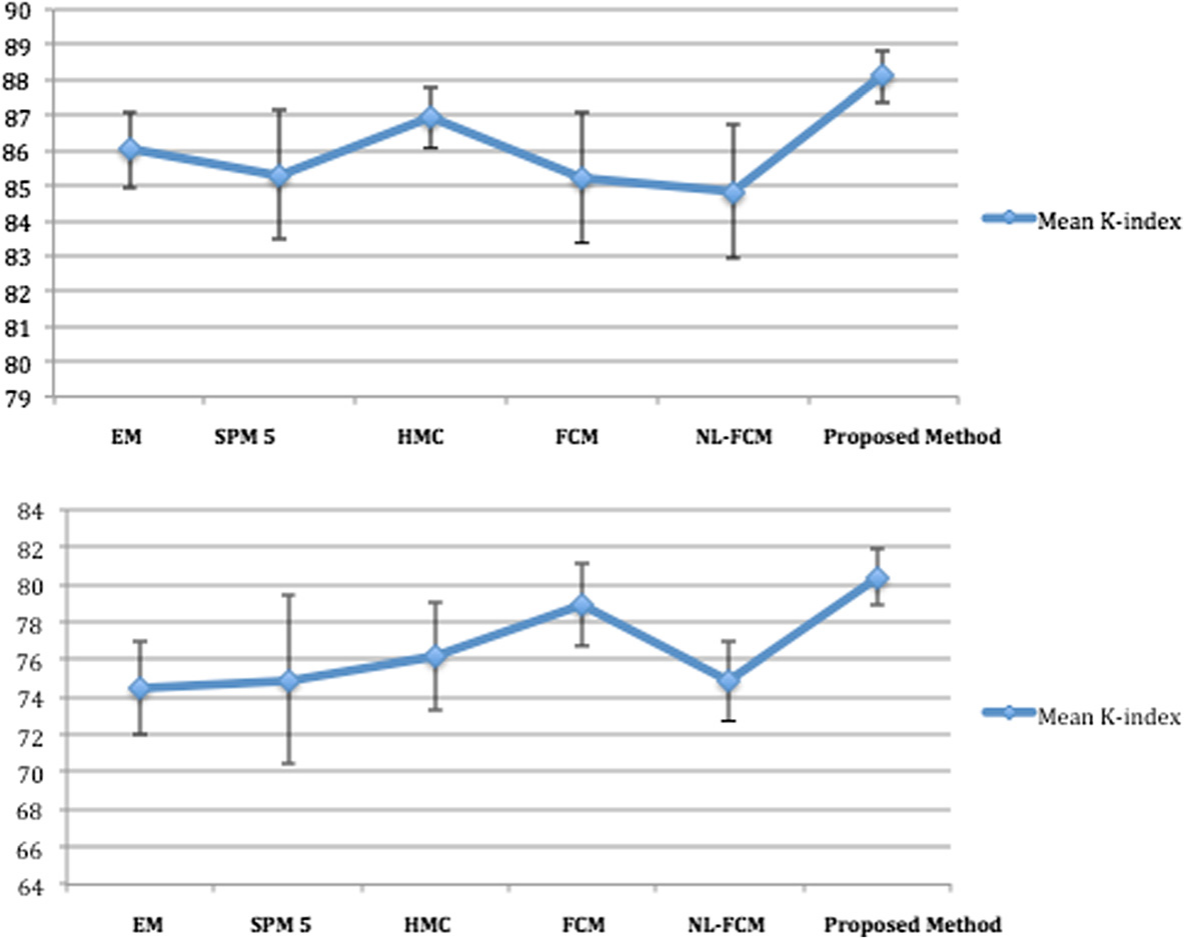
**Mean and standard deviation of K-index for segmentation methods in Figure** **10** **.** Top to bottom: WM graph, GM graph.

As statistical analysis we consider mean and standard deviation of k index for 18 real images and different methods. The accuracy and robustness of methods could be evaluated by the amount of mean and standard deviation. In other words the larger mean leads to more accurate result and the smaller standard deviation leads to robustness (see Figure 11). In this figure the vertical lines indicate the standard deviation and the blue graph presents the mean overlap rate of different methods for 18 real images.

Table 1 and Figure 11 present that the proposed method outperforms other competing algorithms.

In WM segmentation the mean overlap measures of our method is 88.09, which is 2% to 4% higher than other methods. In addition the standard deviation of k-index of proposed method is 1.58, which is 1% to 3% less than other techniques. In addition, in terms of GM segmentation, the results of proposed method are significantly better than WM classification.

Moreover, brain extraction stage may cause differences in the results of brain segmentation in terms of the K-index, as the number of voxels in the segmentation references may vary depending on the brain extraction algorithm.

#### Evaluation with simulated dataset

To point out the contribution of the proposed framework, it would be compared with fuzzy and non-fuzzy algorithms with 20% inhomogeneity and different Rician noise as shown in Table 2.

**Table 2:** **The Kappa index for the WM/GM segmentation of the simulated database with different Rician noise levels and a 20% inhomogeneity**

**Methods**	**White matter (%)**						**Grey matter (%)**				
**Noise level**	**0**	**1**	**5**	**7**	**9**	**0**	**1**	**3**	**5**	**7**	**9**
EM	86.1	91.5	92.2	90.1	86.4	83.1	90.8	92.5	92	89.1	84.2
SPM 5	91.05	94.2	93.6	90.2	86.3	91.2	93.4	93.3	92.1	90	86.6
HMC	97.8	95.7	93.9	937	91.7	97	94.5	95.9	93.8	92.7	91.1
NL-FCM	95.6	94.2	91.5	89.8	83.2	95.4	94.1	93.8	92.9	89.9	79.3
FCM	97.1	96	92	88	84	97	96	91	86	87	83
Proposed-Method	96.8	94.9	93.7	93.9	92.1	95.7	95.4	92.8	92.6	92.1	91.2

The fuzzy techniques are FCM [38] and NL-FCM [20,21] and non-fuzzy algorithms are SPM 5, Hidden Markov Chains (HMC) [37] and EM [36,37]. SPM5 and EM are two free available reference software for MRI classification [35,37].

**Figure 12:**
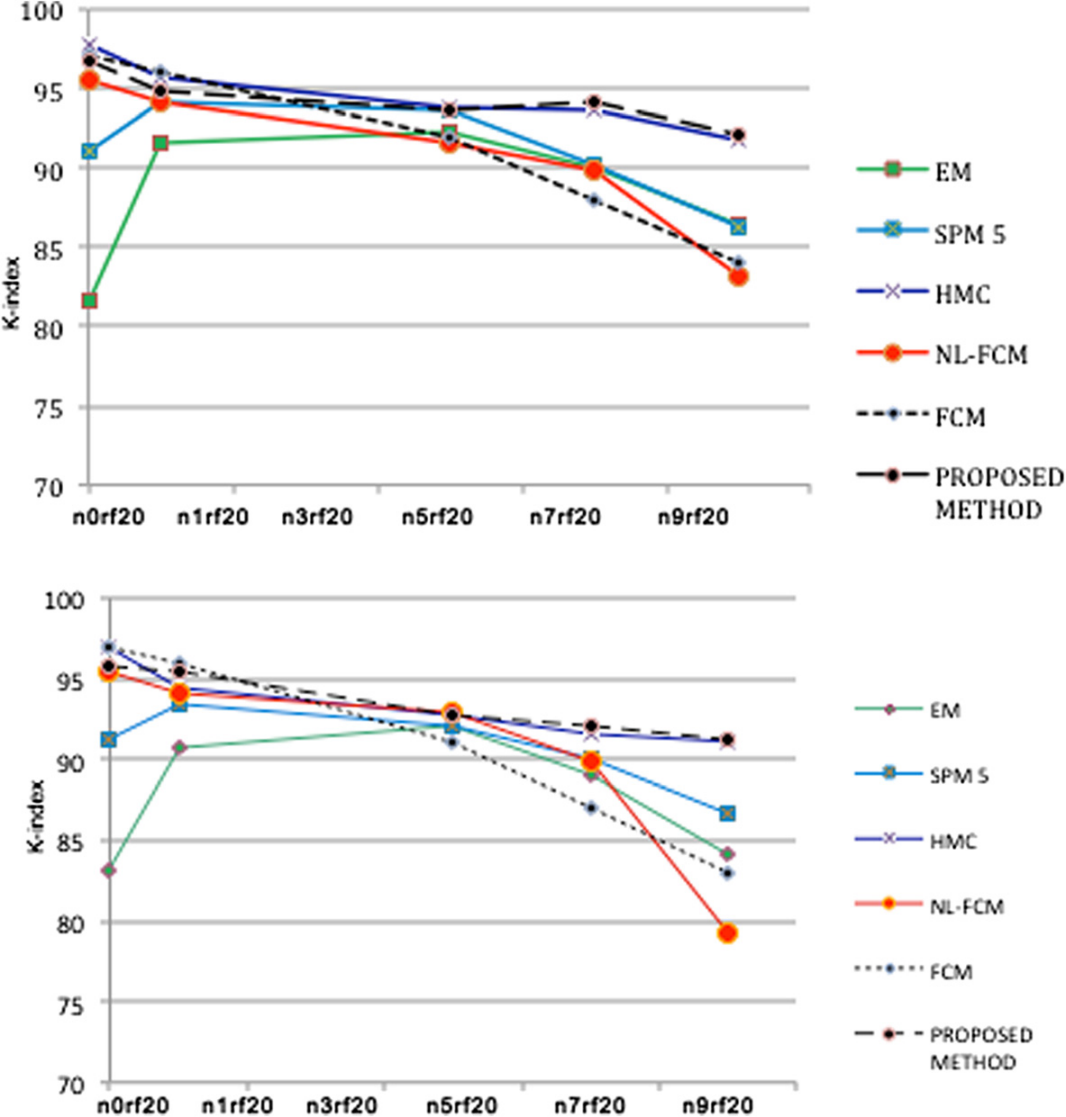
**Application of different techniques on the same simulated image from Brainweb dataset with different Rician noise levels and a 20% inhomogeneity.** Top to bottom: overlap rate (K-index) of WM, Overlap rate of GM. (n presents the level of noise and rf indicates the level of bias field).

We performed experiments with Brainweb database to determine the robustness to noise for the proposed technique. The overlap rate or K-index over the same simulated image in different noise levels and 20% bias field for several algorithms is shown in Figure 12.

The graphs in Figure 12 demonstrate that the proposed framework improves the results. The mean Kappa indexes for WM are: EM =88.36, SPM 5 = 91.07, HMC = 94.30, FCM =91.42, NL-FCM =90.86, and the proposed-Method =94.32. The Average Kappa indexes of GM segmentation are: EM =87.84, SPM 5 = 90.66, FCM =90.8, HMC =93.38, NL-FCM =90.32, and the proposed-Method =93.44.

As can be observed from the graphs, the proposed technique demonstrates relative superiority in comparison with currently used algorithms specifically in noisy images.

The proposed framework also displays satisfactory results in comparison with EM algorithm. In addition presented Markov random chain and FCM algorithms are superior to proposed technique in low-level noise, but in high-level noise our technique is superior. Furthermore Table 2 demonstrates that FCM based methods are not reliable methods for images with high-level noise. Proposed technique also has significant improvement in comparison with SPM method in both GM and WM classification and different levels of noise.

In addition, there are also different free software packages such as SPM, Freesurfer and FSL. Their results are very different depending on what is expected to be obtained. The reason is that these packages have been designed for a slightly different goal, even though they all perform brain tissue segmentation. The differences we have found, both in their performance and their goal, are detailed below [41].

Regarding FSL performance, one of the problems in classifying all tissues is found in the edges between tissues. To solve this problem SVM classifier is used in the proposed method especially for the overlapped regions and brain edge classification. Generally, segmentation result of FSL is heavily influenced by noise, and very little by bias field.

SPM has a better result for WM segmentation when compared to FSL but the result for GM segmentation is slightly worse. Regarding CSF, the result is very poor and cannot be considered if the goal is to segment CSF from a brain image because a part of the scalp and skull are classified as CSF. Our method offers improvement in WM and GM segmentation in comparison with other methods but GM segmentation result has significant improvement.

The performance of SPM package is not influenced by bias field as much as noise, but as like FSL, it is very influenced by noise. The proposed method is less influenced by noise and in high-level noise our technique is superior in comparison with other methods.

Unlike SPM, FSL and our method, FreeSurfer is designed to segment between the brain structures (both cortical and sub-cortical) such as thalamus, hippocampus, etc. FreeSurfer extracts all structures in the brain image, which are composed by white matter and gray matter. The problem is that where SPM, FSL and proposed method diagnose WM or GM or combination of them, FreeSurfer can see a brain structure, such as brain stem [41]. Based on our experience, FreeSurfer package should be mostly applied when we are interested in brain structures instead of segmentation of brain tissues. In comparison with proposed method, FreeSurfer is more affected by noise and bias field and the results of experiments are not satisfactory when the level of noise and bias field are high. The other problem is that FreeSurfer is a registration-based technique. Thus if the registration stage is not performed accurately the results of segmentation degrade considerably.

One of the advantages of proposed method is that it is not directly frdependent on the registration step. In addition in high level noise and bias field the proposed method has robust and more accurate result in comparison with other methods.

In comparison with proposed method, FreeSurfer is the most automatic tool because everything can be performed in one command but the problem is that FreeSurfer tool is more oriented to study of brain anatomy than to identify between WM, GM and CSF [41]. However, if we are really interested in sub-cortical structures, we should use FreeSurfer package as an alternative. If the goal is accurate brain tissue segmentation into WM, GM and CSF, the proposed method is highly recommended.

## Conclusion

In this study, we proposed a novel automatic framework for tissue segmentation in brain MRI by a new combination of Histogram based method, GCM and SVM. The experimental results indicate that the combination of the statistical and the machine learning based segmentation methods can enhance the overall segmentation performance, compared with each component individually. This is because the proposed method takes advantages of the classification ability of machine learning method in addition to the MR intensity and location information, which are consequential information to classify the brain in a 3D MRI into the multiple classes.

Robustness to noise and simplicity are two advantages of proposed framework. The results are independent from registration step and it makes our algorithm faster than other registration-based methods. In addition because our method is designed to run in MatLab, it is not platform-dependent and it can be run in both Linux and Windows operating systems.

In order to investigate the proposed segmentation method, it has been used for brain tissue segmentation using simulated and real data, creating satisfactory results. The experiments demonstrated that the segmentation results are much closer to ground truth.

Experiments on simulated images and real data have indicated that our method obtains higher kappa indexes in most cases compared with other techniques currently in use.

The experiments were first carried out on 20% inhomogeneity and different noise level (1%, 3%, 5%, 7% and 9%) on Brainweb MR scans. These experiments show the robustness and precision of our approach in the presence of inhomogeneity and different levels of noise. Additional experiments carried out on the real data have indicated that this technique reliably extracts brain tissues with accuracy comparable to other currently used algorithms.

The main shortcoming of our method is that in the first stage, which is histogram-based method, we assumed a symmetric Gaussian distribution model for the intensity distribution of brain images. But in real images the estimated Gaussian distribution is not exactly symmetric due to the existence of noise, artifacts and overlapped Gaussians in the histogram. It decreases the accuracy of algorithm about 2-3 percent. Our future work involves improving this shortcoming of the proposed method.

In this paper we used 1.5 Tesla images and compared the proposed method with other methods using the same images. Some of mentioned challenges in this study will be solved by ultra-high MR images. In future work we will consider 3 T images and compare the result with other techniques on the same images.

In terms of application, our method can be helpful in the case of low contrast images. Extension of proposed technique through the modified histogram based technique is the next challenging task for future. In addition the extension of this work for disease and tumor detection could be another challenging work.
